# Supplementary figures and images for: An Oral Botanical Supplement Improves Small Intestinal Bacterial Overgrowth (SIBO) and Facial Redness: Results of an Open-Label Clinical Study
Source: Nutrients. 2024 Sep 18;16(18):3149. doi: 10.3390/nu16183149 (PMC11435404; doi:10.3390/nu16183149)

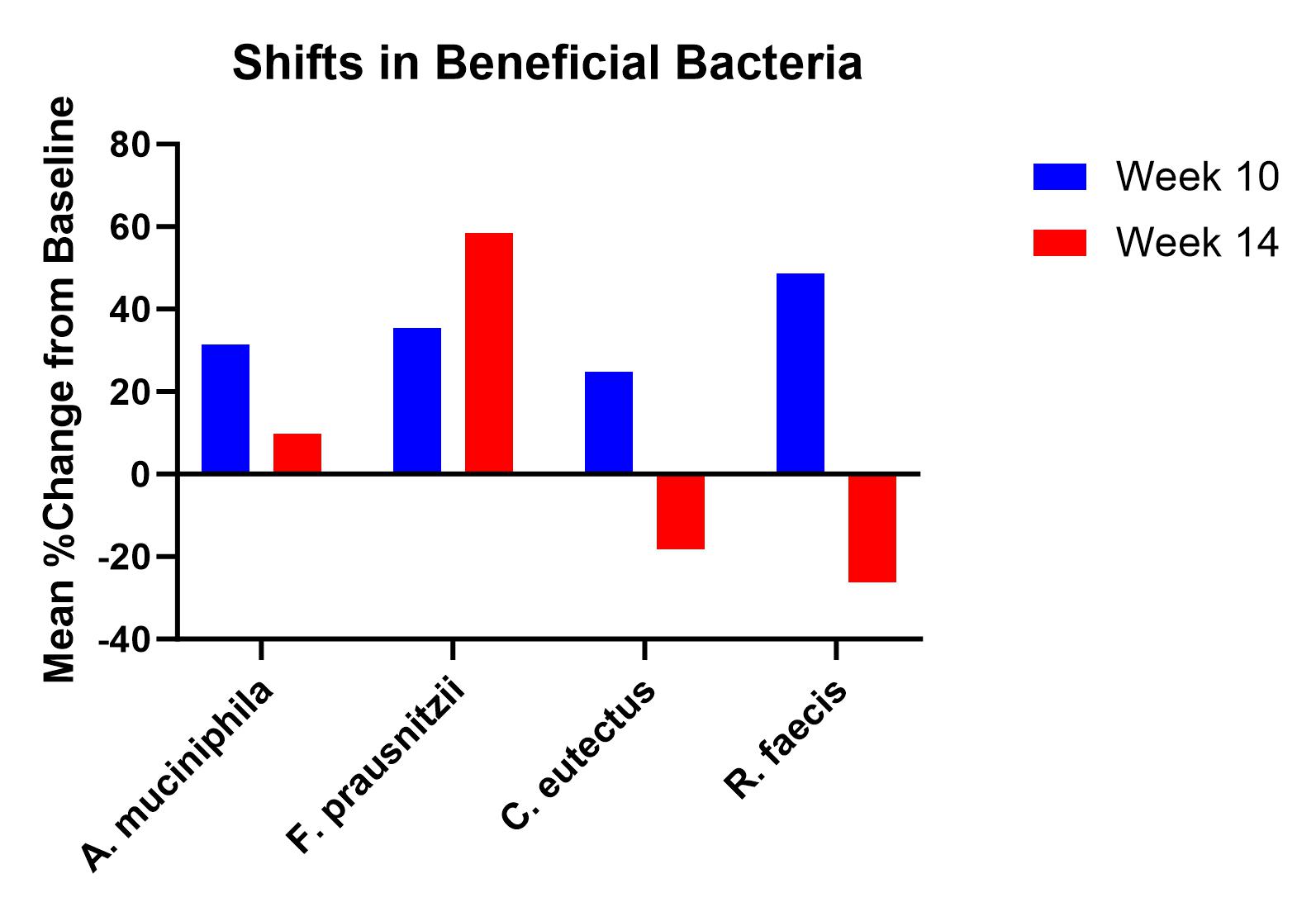

Supplement: Supplementary file 1 [file nutrients-16-03149-s001.zip › FigureS1.jpg]

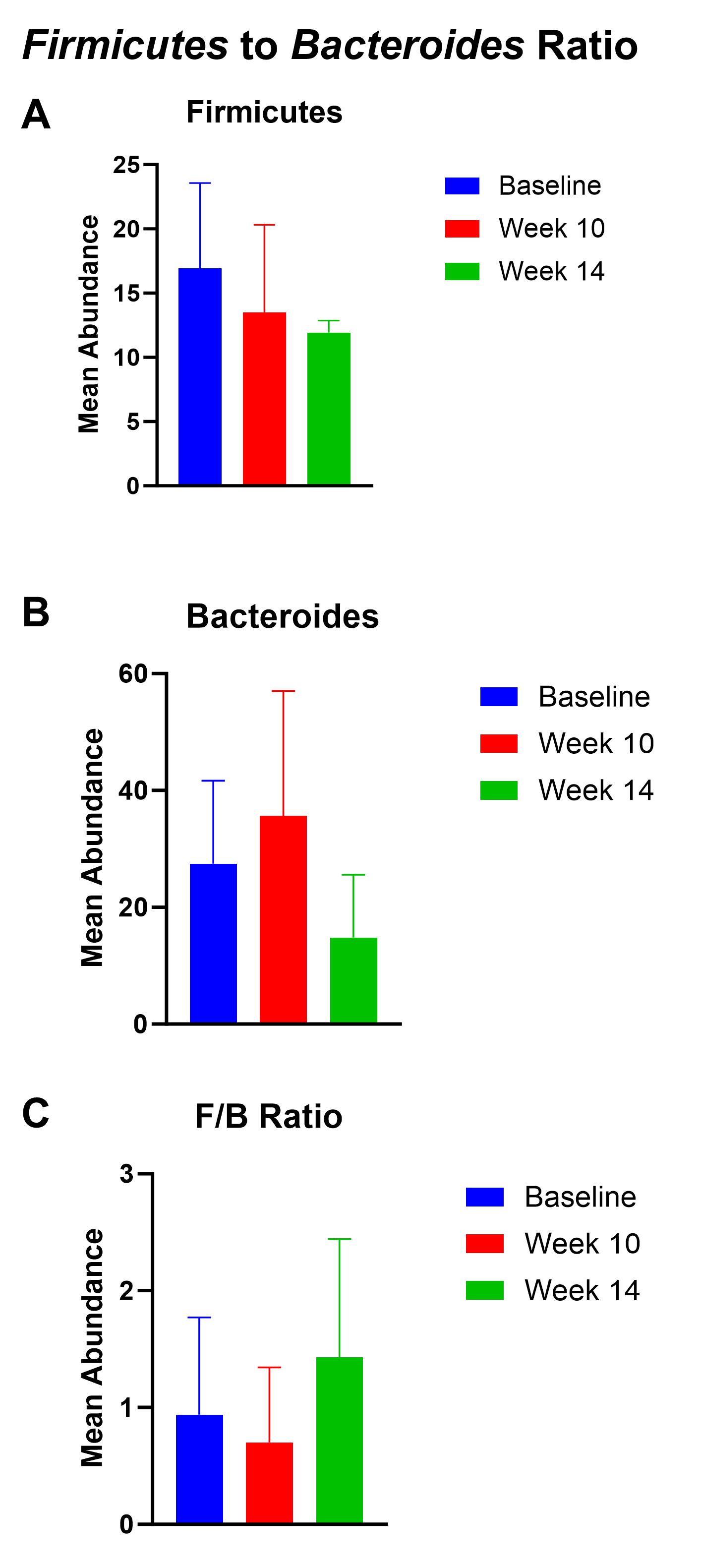

Supplement: Supplementary file 1 [file nutrients-16-03149-s001.zip › FigureS2.jpg]

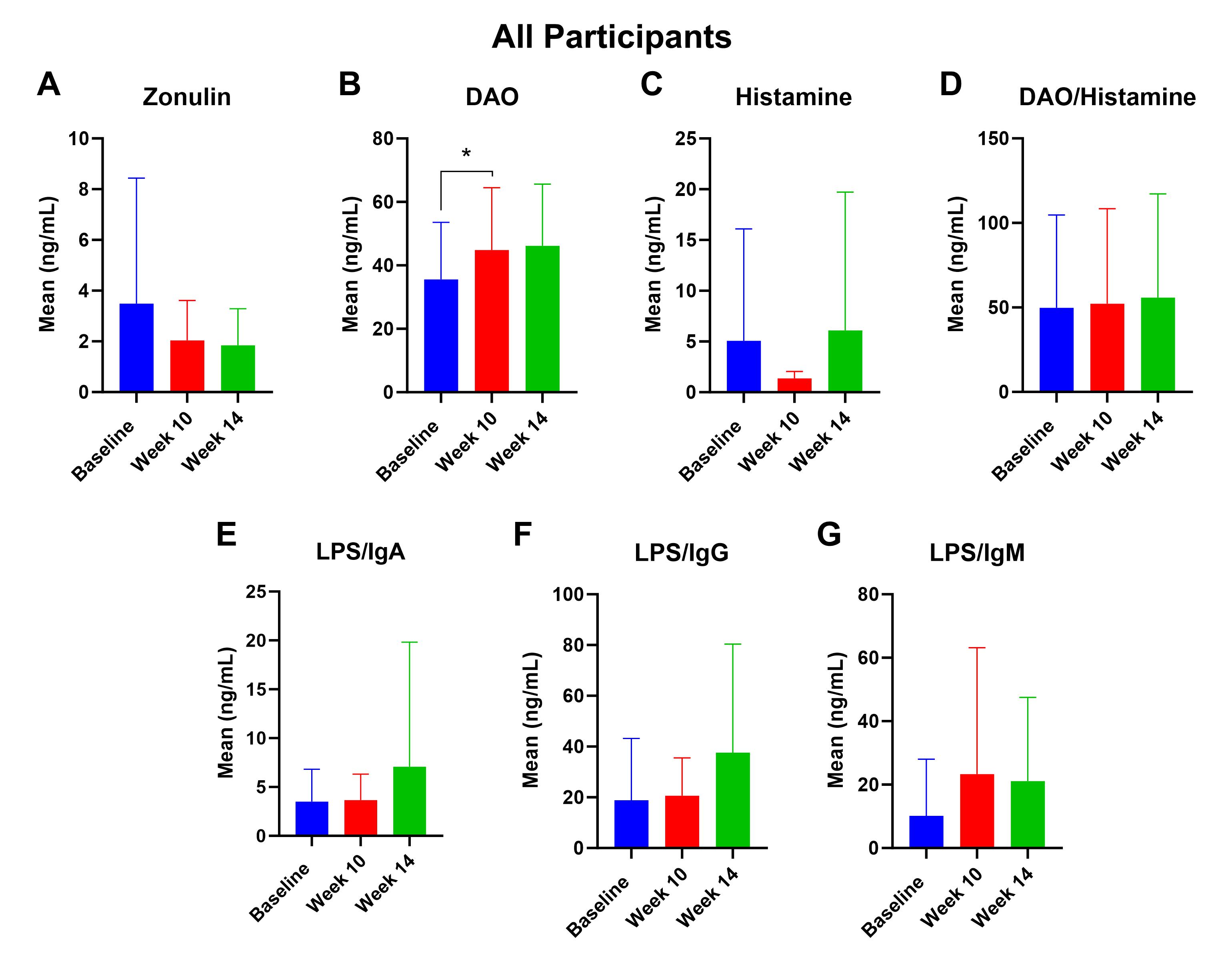

Supplement: Supplementary file 1 [file nutrients-16-03149-s001.zip › FigureS3.jpg]
